# Measuring and evaluating colorimetric properties of samples from loess-paleosol sequences

**DOI:** 10.1016/j.mex.2023.102159

**Published:** 2023-03-31

**Authors:** Christian Laag, France Lagroix, Sebastian Kreutzer, Stoil Chapkanski, Christian Zeeden, Yohan Guyodo

**Affiliations:** a Université Paris Cité, Institut de Physique du Globe de Paris, CNRS, 1 rue Jussieu, Paris, France; b Institute of Geography, Ruprecht-Karl University of Heidelberg, 69120 Heidelberg, Germany; c Archéosciences Bordeaux, UMR 6034, CNRS-Université Bordeaux Montaigne, Pessac, 33600, France; d University of Paris 1, Panthéon-Sorbonne, Laboratory of Physical Geography (LGP), UMR 8591, CNRS, 2 Rue Henri Dunant, 94320, Thiais, Paris, France; e University of Rouen-Normandy, IDEES Laboratory, UMR 6266, CNRS, 17 Rue Lavoisier, 76130, Mont Saint-Aignan, France; f LIAG - Leibniz Institute for Applied Geophysics, Stilleweg 2, 30655 Hannover, Germany

**Keywords:** Colorimetry, Spectrophotometry, Color enhancement, Loess-paleosol sequence, Goethite, Hematite, Calcite, Spectrocolorimetry

## Abstract

Colorimetric measurements are valuable in studying paleoenvironmental changes in sediment archives such as loess-paleosol sequences. These measurements allow for the identification of climate-sensitive minerals such as hematite, goethite, and secondary carbonates, as well as the observation of stratigraphic changes influenced by paleoclimate variations. Herein, a detailed workflow protocol emphasizing mineral abundance extraction by determining true band amplitudes is presented. Moreover, we present a protocol for colorimetric measurements that eliminates container bias, allowing the analysis and re-analysis of stored sediment quickly and inexpensively. Finally, we introduce a new R-package ('LESLIE') for graphical data display and enhancement. The protocol and its validation are demonstrated on the Suhia Kladenetz loess-paleosol sequence of northern Bulgaria.

• A detailed workflow protocol eliminating container bias in colorimetric measurements and extracting mineral abundances is presented.
• The protocol is independently validated with aid of Attenuated Total Reflectance Fourier Transform mid-infrared (ATR-FTIR) spectroscopic experiments.
• Stratigraphic color enhancement using the R-package 'LESLIE' is facilitated by a user-friendly R-shiny application.

## Graphical abstract

**Figure:**
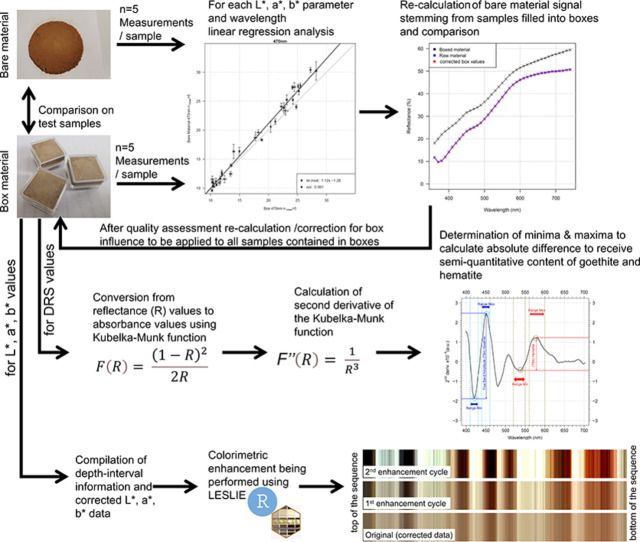

Specifications table

**Table:** 

Subject area	Environmental Science

More specific subject area:	*Spectrocolorimetry*
Name of your method:	*Spectrocolorimetry*
Name and reference of original method:	C. Zeeden, L. Krauß, F. Lehmkuhl, H. Kels, Supplementary R script for manuscript “Digital image analysis of outcropping sediments: comparison to photospectrometric data from Quaternary Loess deposits at Sanovita (Romania) and Achenheim (France),” (2016). https://doi.org/10.5880/SFB806.17. T. Sprafke, P. Schulte, S. Meyer-Heintze, M. Händel, T. Einwögerer, U. Simon, R. Peticzka, C. Schäfer, F. Lehmkuhl, B. Terhorst, Paleoenvironments from robust loess stratigraphy using high-resolution color and grain-size data of the last glacial Krems-Wachtberg record (NE Austria), Quaternary Science Reviews. 248 (2020) 106,602. https://doi.org/10.1016/j.quascirev.2020.106602. Z. Jiang, Q. Liu, A.P. Roberts, M.J. Dekkers, V. Barrón, J. Torrent, S. Li, The Magnetic and Color Reflectance Properties of Hematite: From Earth to Mars, Reviews of Geophysics. 60 (2022). https://doi.org/10.1029/2020RG000698.
Resource availability:	https://doi.org/10.5281/zenodo.7257766.

## Method details

### Measurement of reflectance from infrared to visible light spectra

Colorimetric measurements within the visible light range (400–700 nm) performed on sediments such as loess-paleosol sequences are inexpensive and straightforward to apply. Paleoenvironmental studies increasingly report colorimetry data providing a powerful complement to routinely presented environmental magnetism or granulometric analysis [1], [2], [3]. Contrarily, magnetic measurements discriminating hematite and goethite, which are important indicators of past climate conditions, are time-consuming. Determining quantitative and absolute concentrations is challenging because of their relatively weak magnetizations and their inherent grain-size dependence on most magnetic properties. Paramagnetic and diamagnetic (i.e., non-remanence bearing) silicate and carbonate minerals such as calcite are not identifiable, and concentrations are non-quantifiable via magnetic analytical methods. It has been demonstrated [4], [5], [6] that colorimetric measurements can estimate, semi-quantitatively, amounts of goethite and hematite and that luminance (L*) can proxy, qualitatively, for amounts of calcite. Thus, colorimetric measurements provide an economical and non-time-consuming alternative to other methods.

Colorimetric measurements with spectrophotometers present many advantages over retrieving color codes from Munsell color charts. Munsell color observations are highly subjective, dependent on the light source, the observer's color perception, and sensitivity to slight differences in color-nuances. Moreover, the Munsell color system is non-continuous, urging the researcher to sort samples into color classes, inevitably leading to lower resolution and data loss.

The past decades of loess research underlined the clear benefits of examining colorimetric measurements to gain a deeper paleoclimate understanding of the archive studied [2,^3^,[7], [8], [9], [10]]. Spectrophotometers measure color with the aid of different standard illuminants, for example, A, D50, C, or D65 (used in the present study) inside the CIE1931 color space. Raw colorimetric reflectance data, usually acquired between 400 nm and 700 nm in 0.5 nm to 10 nm intervals, is transformed with the illuminant specific intensity at a distinct wavelength into tristimulus values, $\bar{x},\bar{y},\bar{z}$ dependent on the observer angle (2° or 10°). From these spectra, values positioning the colorimetric measurement in a three-dimensional color space (ISO/CIE 11,664–4:2019) are calculated. The defined color-sphere has a vertical y-axis (luminance value L*) ranging from 0% (total black) to 100% (total white), an x-axis (redness value a*) with undefined limits ranging from negative (green) to positive (red), and a z-axis (b*) with undefined limits ranging from negative (blue) to positive (yellow).

### Instrumental settings for DRS-VIS measurements

All colorimetric measurements of the visible light range (VIS) were performed with a Konica Minolta CM-600d spectrophotometer. Before measurements, the spectrophotometer was calibrated with Konica Minolta-provided white and black standards. The resolution of Diffuse Reflectance Spectroscopy (DRS) data is 10 nm in the visible light range from 400 nm to 700 nm. The measurements were performed with a 0.8 cm wide open oculus, a D65 norm light source, the specular component SCE, and an observer angle of 2° (CIE 1931 standard observer angle). Each sample was measured five times to calculate the mean and standard deviation.

### DRS measurements performed on bare material

**Fig. 1:**
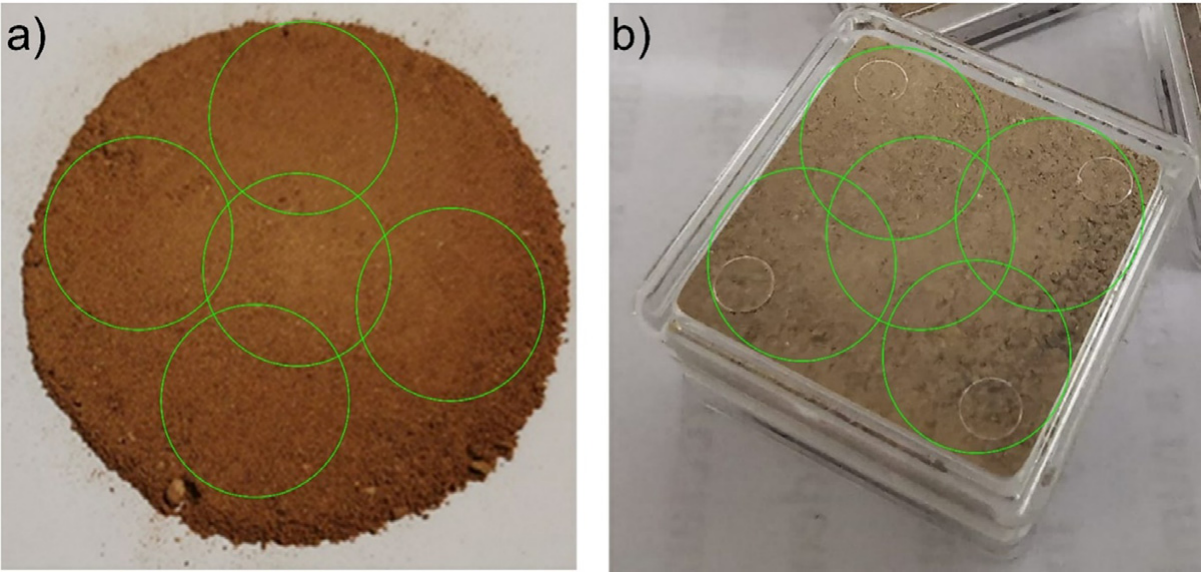
The five measurement targets followed for a) raw material and b) boxed material.

For measurements carried out on bare material, approximately 20 g of material was placed on white paper and compressed with a massive (2 kg) plain surface stainless steel cylinder to avoid shadows and to simulate the compaction inside the plastic boxes. The five measurements were acquired at different positions of the compressed sample material (see Fig. 1a). Each time, the sample material was re-mixed and compressed again with the stainless-steel cylinder to avoid user-induced cracks of the material, which would lead to artificially lower L* values due to shadows between non-compressed material. Only raw data was extracted from the Konica Minolta CM-600d device using the Konica Minolta *SpectraMagix™* software. Raw data consists of reflectance values from 400 to 700 nm in 10 nm intervals and device-internally calculated L*, a*, b* values. Averages and standard deviations from 5 performed measurements per sample were calculated in R [11] and exported in a summary table.

### DRS measurements performed on boxed material

Colorimetric measurements may not have been conducted in an original study of a loess and paleosol sequence, which commonly prepares and stores the sediment in plastic boxes. In many circumstances, removing the sediment to perform subsequent colorimetric measurements would be unfavorable. Paleomagnetic and magnetic fabric studies require samples to have a geographical reference frame. Removing them from their orientated containers would most likely lead to loss of orientation and/or destruction of the poorly consolidated bloc of a sample. For other studies, a limited amount of sample material may have been taken during fieldwork, and just enough to fill a standard (for environmental magnetic studies) size plastic box (e.g., 2 × 2 × 2cm^3^). Opening each sample box (sometimes thousands) to conduct colorimetric measurements on bare material and refilling into the boxes is very time-consuming and leads to unavoidable sample material loss. Since mass normalizing magnetic properties is best and standard, a material loss would require additional work of re-measuring sediment masses. Multiple sub-sampling of a single sample in multidisciplinary studies tends towards a similar issue of limited available material. For the various situations above, there is a real necessity for through-the-box colorimetric measurements.

**Fig. 2:**
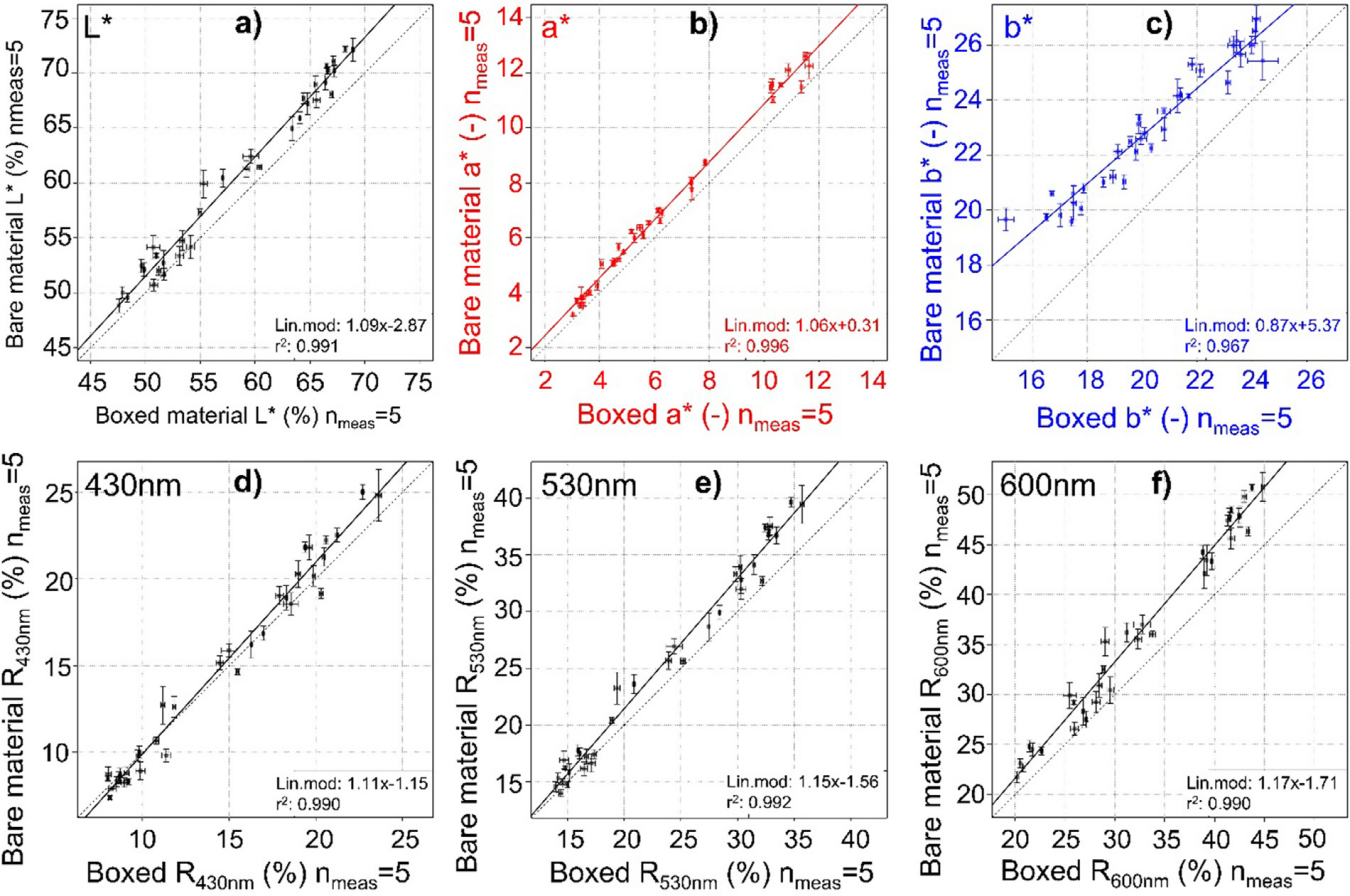
Comparison of 35 samples measured through the box (abscissa) and on raw material (ordinate). L*, a*, and b* values are shown in a)-c) and examples of three wavelengths (430 nm, 530 nm, and 600 nm) in d)-f)). Standard deviations resulting from 5 measurements are shown as error bars. In each figure, the solid line is the linear regression model of the dataset, and the dotted line is the 1:1 ratio with a slope of 1. Linear model parameters are given together with r^2^ in the legend on each graph at the bottom right and reported in Table 1.

Measurements through the boxes were carried out at five different positions (see Fig. 1b). The experiment was performed under day-time independent laboratory room light sources and on material that was homogenized, carefully hand-grinded, and dried at room temperature for several weeks; the later to avoid the influence of water content (decreasing, e.g., L*). The comparison between reflectance data acquired on a given sample from measurements performed through the plastic box and the bare material was compared. Afterward, this data was plotted for each wavelength in biplots (see Fig. 2d, e, and f for examples at 430 nm, 530 nm, and 600 nm, respectively) to investigate their correlation and dependency. Linear models were calculated, and linear regression functions were determined and listed in Table 1.

**Table 1:** Linear model parameters and their correlations of the selected 35 samples were measured through the plastic boxes and on raw material. Blue and red-shaded table entries highlight the range of wavelengths for goethite (blue) and hematite (red) over which minimum and maximum were searched.

Thirty-five sediment samples of the 1136 sampling depths from the Suhia Kladenetz (SK18) loess and paleosol sequence (LPS) near Pleven in northern Bulgaria were selected. For a detailed description of the SK18 LPS, please refer to Jordanova et al. (2022) [12]. The samples selected for this study represent a mixture of end-member colorimetric values such as high and low a*, high and low L*, and high and low b* to build robust regression functions. The boxes are made of non-magnetic ultra-clear plastic (Caubère reference 221 (19×19×10 mm, 4 cm^3^), material: crystal-clear polystyrene PS with a thickness of 1 mm) and were stored using layer-wide wipes to avoid scratches from non-consolidated sample material. The bare sediment to boxed sediment comparison regression functions should be considered plastic box brand and type dependent. The correction method developed in this study can be followed for other brands and types of plastic boxes.

### Comparison of boxed and bare material

The first analyses evidenced that the boxes’ colorimetric signal is not a fixed absolute value to be added or subtracted from the measured signal. Therefore, we calculated for each parameter (L*, a*, b*) and all individual DRS wavelength-reflectance values single regression functions to eliminate the box signal.

Fig. 2 demonstrated that not all colorimetric regression parameters between boxed and bare materials are identical. Nevertheless, regressions are linear.

**Fig. 3:**
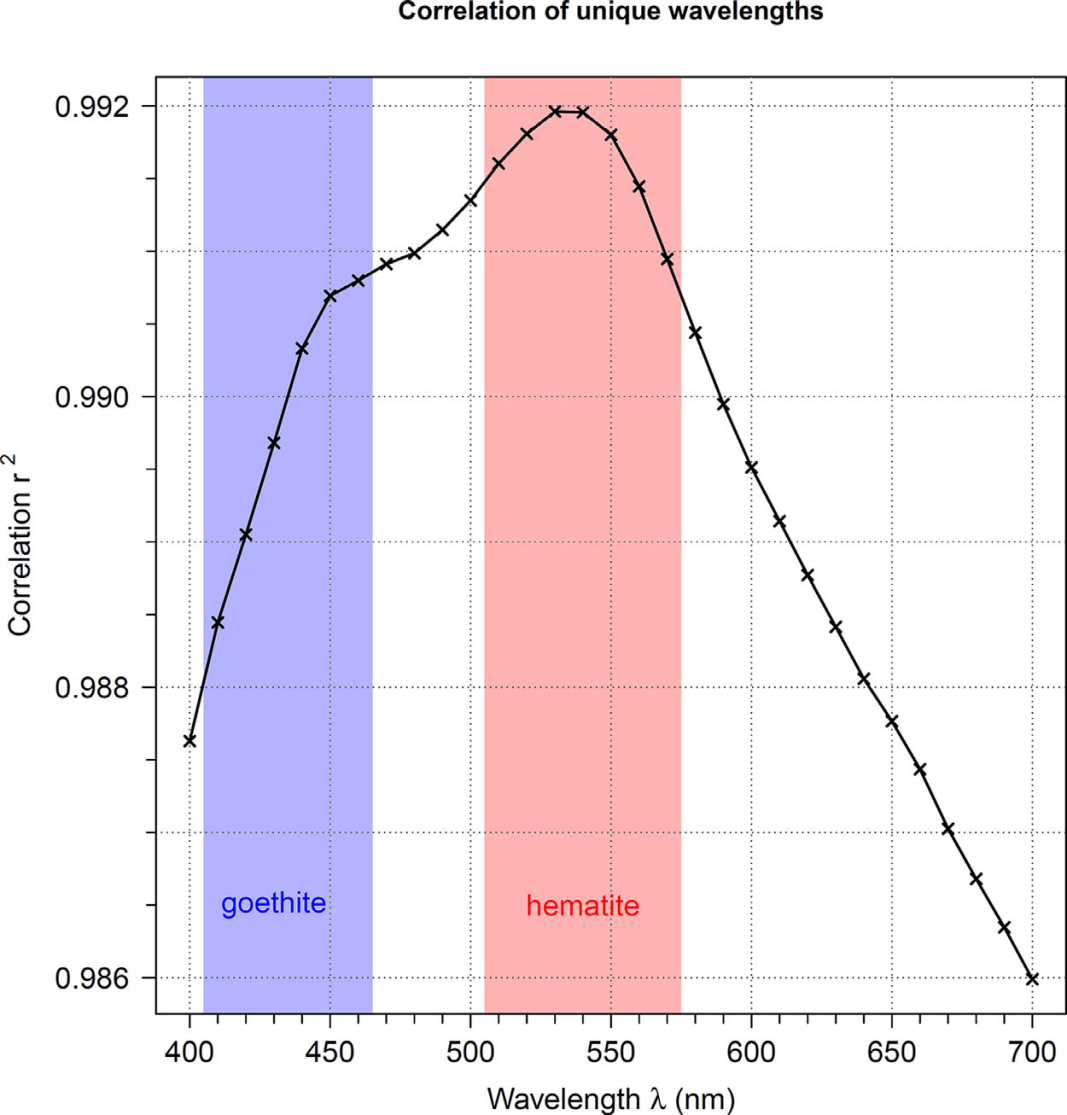
Compilation of correlation coefficients for each wavelength. Wavelength intervals from which goethite and hematite TBA are extracted are highlighted in blue and red.

Each regression function was then applied to the corresponding raw parameters to adjust the DRS spectra to represent bare material measured through a plastic box. A visual confirmation of validity is shown in Fig. 3.

## Reflectance to absorbance transformation

In the next step, we transformed the DRS parameters from reflectance to absorbance values employing the Kubelka-Munk function [13] given from

$$F\left(R\right)=\;\frac{{\left(1-R\right)}^{2}}{2R}.$$

*R* is the dimensionless (recalculated to decimal values since Konica Minolta values are provided as percentages) relative reflectance value (provided by Konica Minolta Instruments). We investigated all correlation parameters for each DRS variable (Fig. 3, Table 1) to ensure that the investigation intervals for hematite and goethite are well-defined and robust (see Table 1).

### Extraction of semi-quantitative goethite and hematite concentrations

Subsequently, the second derivative of the DRS data was calculated following [14], [15], [16], and the absolute and true band amplitude (TBA) was identified following [7]. The TBA is the absolute peak height in the second derivative of the Kubelka-Munk (KM) absorbance transformed reflectance values and estimates the semi-quantitative abundance of goethite and hematite. We considered the first minimum of the second derivative of the KM-recalculated absorbance of reflectance data for goethite between 410 nm and 430 nm and the maximum between 440 nm and 460 nm. The absolute difference of these reflectance values in the ordinate of the second derivative of the DRS data indicates the semi-quantitative amount of hematite and goethite [14,^17^] determined for each of the five individual measurements of each sediment sample.

The same procedure was applied to estimate semi-quantitative hematite content for each sample and its five measurements. A minimum of the second derivative absorbance spectrum between 510 nm and 530 nm and a maximum between 550 nm and 580 nm were identified to calculate the hematite TBA.

Our procedure to determine the TBA by searching a range of wavelengths to identify maximum and minimum deviates strategically from the commonly applied procedure of determining TBA from fixed wavelength values. Cation substitutions (e.g., aluminum) and particle size of hematite and goethite are known to shift DRS peak positions [17,^18^]. Varying natures of hematite and goethite in LPS are expected, given that species of detrital, pedogenic, and diagenetic origins co-exist at the scale of the LPS and may co-exist at the scale of a sample. Therefore, a wavelength interval-based selection of absorbance values rather than a fixed value is justified.

### Recalculating true color DRS values from boxed measurements

Scatterplots of bare and boxed material measurements (Fig. 2) indicate all individual wavelengths' assumed linear behavior, allowing the application of linear regression models. Correlation coefficients (Fig. 3) indicate an overall excellent agreement, with the lowest correlation coefficient at *r* = 0.986 by correlating box and bare material measurements of wavelengths at the end of the spectrum (690 nm and 700 nm) and the highest correlation coefficients at *r* = 0.992 for the wavelengths of 510 nm to 550 nm (Fig. 3). The scanning intervals for goethite (taking minimum and maximum into account) from 410 nm to 460 nm range with their *r* = 0.988 to *r* = 0.991, for hematite (between 520 nm and 580 nm) between *r* = 0.990 and *r* = 0.992 (Fig. 3).

The slopes of the linear models were always positive (>1) with a minimal negative y-axis intersection (Table 1). Slopes differ minimally for wavelengths in the lower end of the spectrum (400 nm and 410 nm) but remain > 1, whereas the highest slopes are reached around 550 nm and 580 nm. The mean and standard deviations of bare-material measurements are generally slightly higher than those calculated for boxed material. This observation may be due to the spreading/cracking of the compressed bare material (increasing mean and standard deviation) or a higher overlap of measurement areas of the boxed samples (decreasing mean and standard deviation), as shown in Fig. 1.

**Fig. 4:**
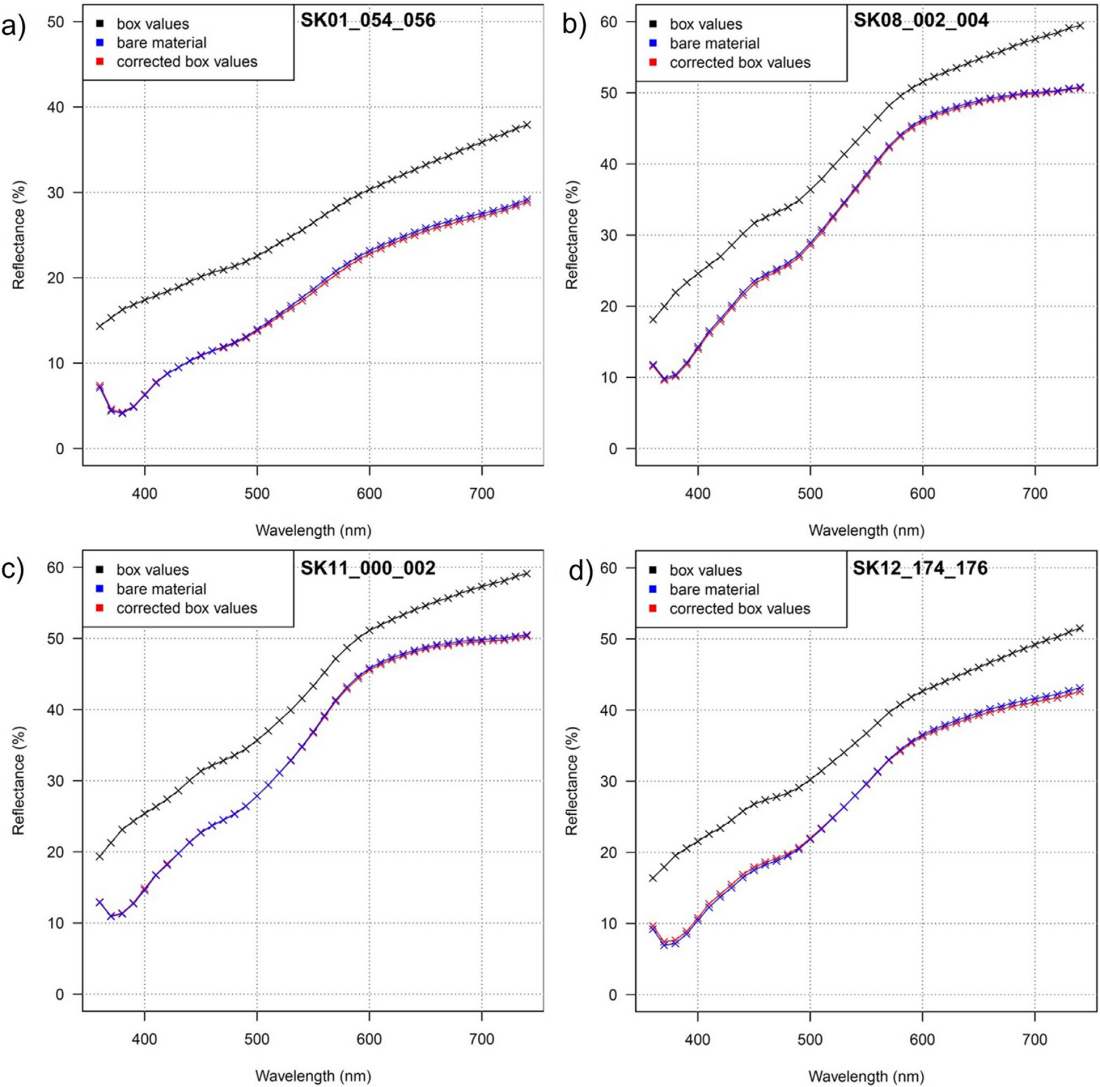
Comparison of measured DRS spectra on boxed material (black) and bare material (blue) and the calculated (corrected) DRS spectra of the boxed material of 4 of the 35 representative samples of the SK18 LPS.

From the calculated linear models for each wavelength, a comparison was carried out between bare material, box material, and re-calculated bare material. In Fig. 4, all three spectra are plotted and compared. Fig. 4 shows the successful recalculation of the raw material from material contained in the boxes for four of the 35 samples.

### Color contrast enhancement

Our contrast enhancement algorithm is motivated by two ideas presented in Zeeden et al. [19,^20^] (color values presented rectangles in stratigraphic order) and Sprafke et al. [21] (contrast enhancement) but seeks to simplify and strengthen the outcome. Zeeden et al. [19,^20^] provided an R-Script to represent colorimetric values as rectangles in stratigraphical order. Sprafke et al. [21] used contrast enhancement based on *MS Excel*^*TM*^ to identify cold and warm periods, represented by rather “cold” and “warm” colors. Here, we combine both approaches to provide an enhanced stratigraphic overview of an LPS in a documented R package called 'LESLIE' (LoESs coLorimetry sIgnal Enhancement) [22] designed to re-calculate the combined L*, a*, b* values to RGB values and to perform a user-defined number of enhancement cycles. The R package and it's example data allow the user to adjust the data structure for the R package using the given example (prepare your data with depths in one column and L*, a*, b* values in columns 2–4). Depth limits are user-defined or are calculated automatically if no depth interval for each sample is given. For usability reasons, we limited the user interaction to selecting a file, the number of enhancement cycles, and a few graphical adjustments parameters. The number of suitable enhancement cycles is subjective. We recommend two to three enhancement cycles, which we consider sufficient. However, users may want to test with more enhancement cycles. The re-calculated and enhanced values are returned as numerical values to be exported as comma-separated files (CSV), among other possible formats, and used in different applications. 'LESLIE' also includes a simple ‘shiny’ [23] based web interface for users less familiar with R. The application can be launched by typing LESLIE::app_LESLIE() inside the R terminal.

### Integrating color enhancement and box-correction

**Fig. 5:**
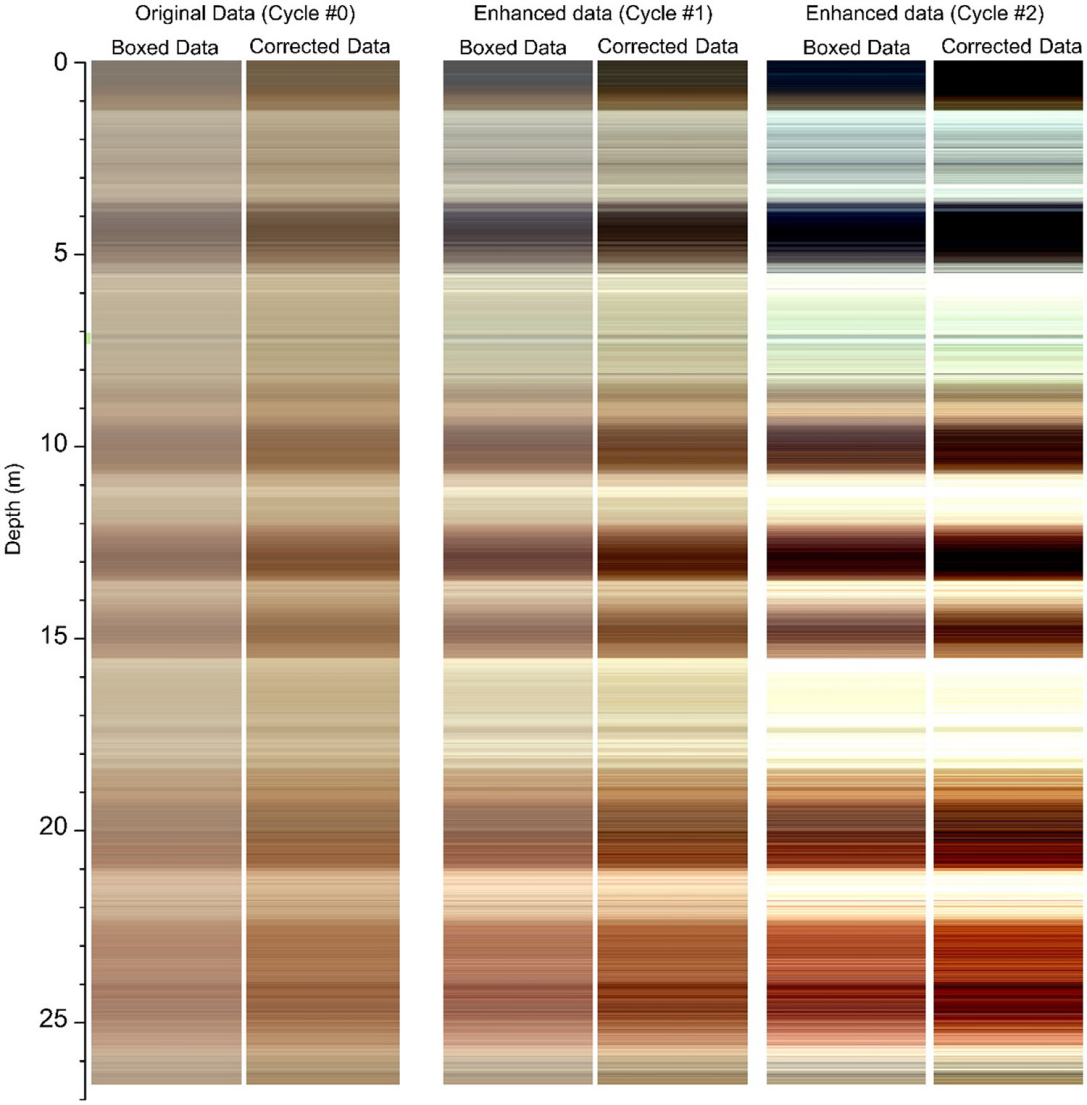
Compares the applied colorimetric enhancement cycles performed on the SK18 LPS (1336 samples) boxed material before and after correction. Note that the original data of the uncorrected box material (leftmost panel) are RGB values calculated from the average L*, a*, and b* values of 5 measured DRS-spectra for each of the 1336 samples (i.e., a total of 6680 individual DRS-spectra measurement).

A comparison between the uncorrected (boxed material) and the corrected/recalculated spectra following the colorimetric enhancement protocol is shown in Fig. 5 for two enhancement cycles of analyses obtained on the SK18 LPS's 1336 samples. Fig. 5 shows, for each cycle, L*, a*, b* values were re-calculated as RGB values for the boxed sample before correction (left panels) and after correction (right panels).

The resulting RGB colors for the non-corrected L*, a*, b* values are dim or muted, leading to less visible color changes in the stratigraphic plot.

The right-panel figures of each set of sub-plots have a more contrasted and sharp stratigraphic differentiation. Transitions from one stratigraphic unit to another are more easily identifiable. Thus, the colorimetric enhancement (cycle #0 being the original data and cycles #1 and #2 the enhanced values) adds value to the investigator's visual interpretation of the color column. In cycle #2 of the corrected data, one can observe a better-expressed reddening in the lower half of the stratigraphic column than in the original (corrected) data. This reddening was already observed in [12].

## Method validation via ATR-FTIR

Mid-infrared spectrometry, capable of tracking goethite and calcite concentrations semi-quantitatively, was measured on a subset of 719 samples from the SK18 LPS using a Fourier Transform Infrared (FTIR) Brucker Vector 22 spectrometer equipped with an Attenuated Total Reflection (ATR) sampling accessory and diamond crystal. The samples (mean of 64 scans) were scanned from 4000 cm^−1^ to 400 cm^−1^ at an 8 cm^−1^ resolution. Using the substation between minima and maxima of wavenumber absorbance, two band peak values were selected to calculate the mean signal value for calcite and goethite. Wavenumbers were selected after [24] for goethite (450 cm^−1^) and [25] for calcite (1428 cm^−1^). For calcite, note that the laser beam activates the stretching mode of CO_3_ in an asymmetrical behavior [26]. This causes a strong peak in the absorbance spectrum calculated from the ATR-FTIR measured spectra. However, the resulting peak wavenumber-position at 1428 cm^−1^ is experimentally considered and is accompanied by a standard deviation of ± 11 cm^−1^. We tried to circumvent this possible deviation from a fixed wavenumber position by applying ranges of the absorbance signal's minimum- and maximum wavenumber position per sample, allowing for a standard deviation that does not influence the calculated absolute band amplitude.

The ATR-FTIR semi-quantitative concentrations for goethite and calcite were compared to the corrected box DRS goethite TBA and luminance L* values. Calcite (in primary and/or secondary form) is a very bright, nearly white mineral, which clearly influences the overall luminance (L*). Previous LPS studies, e.g., [20], have demonstrated calcite content to correlate well to L*. Moreover, during field sampling of SK18 LPS, we conducted systematical tests with low-concentrated HCl at each sampling depth and attested to the occurrence of carbonates (see supplementary Table 1S of [12]). Therefore, DRS-derived L* values are considered here to proxy for calcite concentration.

**Fig. 6:**
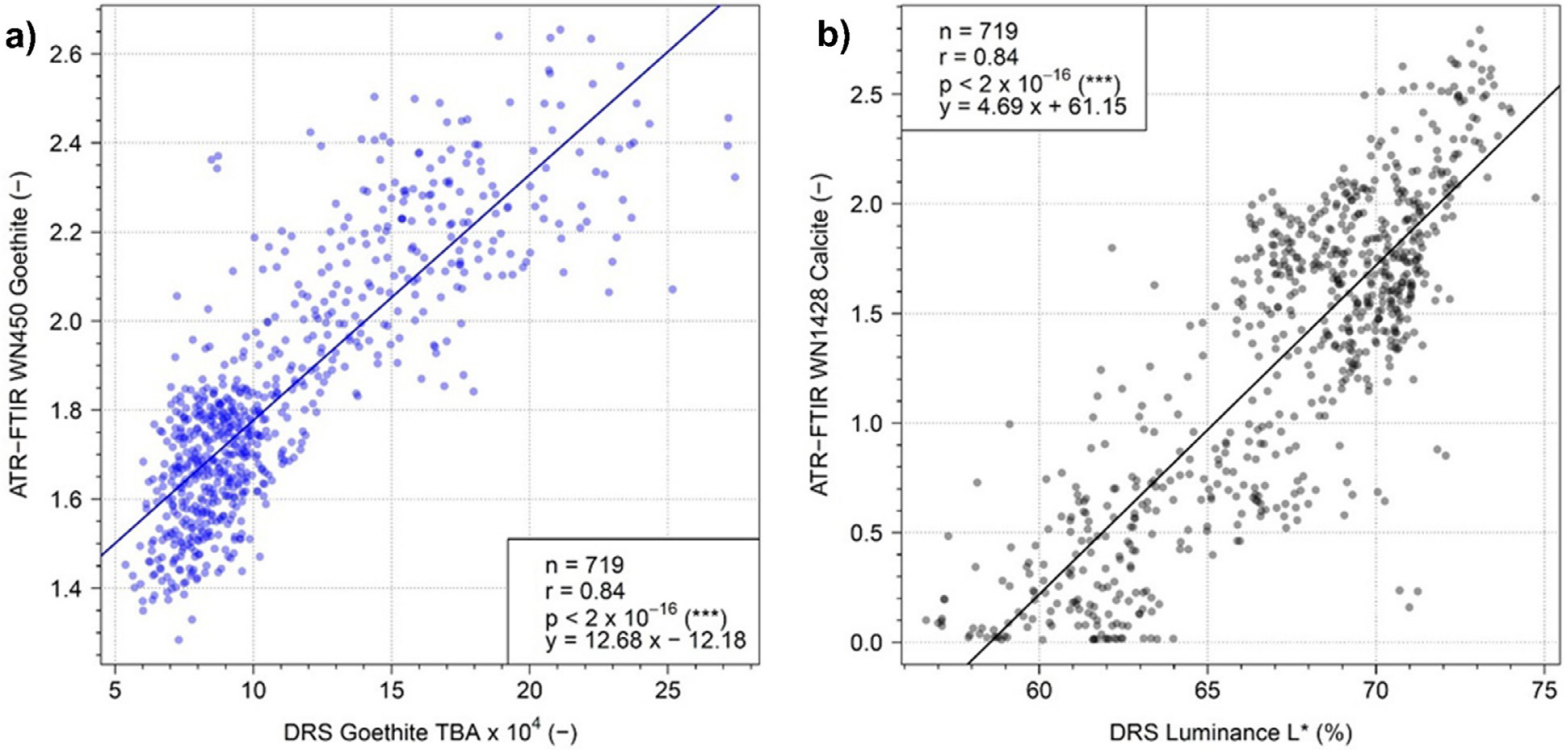
a) Comparison of DRS-derived qualitative goethite content from corrected box data and ATR-FTIR-derived goethite content. b) Comparison of DRS-derived L* values from corrected box data, considered here as a proxy of calcite content (see text), and ATR-FTIR-derived calcite content. Based on 719 data points, both comparisons follow a linear trend and correlate significantly well.

Fig. 6 shows the relationship between the two independent methods (DRS-derived goethite and luminance values and ATR-FTIR-derived goethite and calcite values) for estimating semi-quantitative goethite and calcite concentrations. The linear correlation coefficient between the two different analytical methods is good, *r* = 0.84 for goethite concentration comparisons (Fig. 6a). The significance of the correlations, evaluated from an F-test, is excellent (F value: 1672.8 at 717 degrees of freedom), with *p* < 2 × 10^−16^. A linear correlation observed for the DRS-derived luminance (L*) and ATR-FTIR-derived calcite content comparison (Fig. 6b) is also good (*r* = 0.84). The significance of the correlation is also excellent, with *p* < 2 × 10^−16^ (F value: 1703.9, at 717 degrees of freedom).

## Additional information

Colorimetric measurements and investigation of diffuse reflectance spectra are valuable experiments to gain quick information about the semi-quantitative contents of calcite/dolomite, hematite, and goethite. All these minerals play an important role in paleoclimatic reconstructions performed on loess-paleosol sequences because of their sensitivity to environmental and climate conditions necessary for their formation and preservation. Identifying these minerals via magnetic methods at the scale of an entire LPS can be costly and/or time-consuming but possible. While magnetic methods are highly sensitive, having detection limits for magnetically ordered mineral species on the order of 1 ppm, a limiting factor is its inability to provide absolute contents of goethite, hematite, and calcite/dolomite. XRD analysis can be quantitative for a large set of minerals in sufficiently high concentrations, and quantification methods are proven reliable. However, XRD analyses are time-consuming and costly. The analysis of an entire LPS with thousands of samples by XRD is economical non-viable and unrealistic.

Similar to magnetic methods and even more limited is the restriction of colorimetric methods to 2–4 mineral species based on the resolution of broadly used spectrophotometers. These obstacles in reaching absolute contents could be tackled by coupling the DRS method as described here with XRD analyses with the set goal to experimentally establish reliable and statistically robust transfer functions allowing the absolute quantification of hematite and goethite contents preserved in investigated sediments. Hence, the purpose of this contribution can be split into three parts yielding an overall improved protocol to perform and evaluate colorimetric data.

**Preparation:** the first part contains the sample material preparation and the experimental setup. A detailed protocol is presented which leads, even with rather coarsely resolved diffuse reflectance spectra (10 nm resolution in the visible light wavelength (λ) interval 400–700 nm), to a robust identification of hematite and goethite which are both valuable proxies for precipitation, temperature and seasonality which lead to different weathering intensities of sediments. The derived parameters from DRS spectra for luminance (L*), redness (a*), and blueness (b*) provide in a case for L* and a* reliable proxies of weathering intensity. When L* is decreased (darker), it rather indicates an enhanced abundance of, e.g., weathered organic matter. With increasing L*, the abundance of calcite and dolomite increases, as indicated in this study by the good positive correlation of ATR-FTIR derived calcite and dolomite contents to L* values. The DRS-derived goethite content was thus validated with the aid of ATR-FTIR-derived goethite semi-quantitative contents.

**Remove biasing effects:** the second part targets eliminating a biasing effect of polystyrene boxes, in which sedimentary samples are commonly stored for diverse reasons discussed earlier. The presented protocol removing the biasing effect of measuring DRS spectra through plastic containers unleashes the possibility for many laboratories to investigate or re-investigate stored samples and gain additional information about past climate change without conducting further field campaigns. These box-induced biasing effects significantly reduce the diminished color signal, as presented in Fig. 5. The elimination process enables the reconstruction of the DRS spectrum for each sample as it would be measured without the box. Retrieving reliable DRS spectra leads to a more robust semi-quantitative estimation of goethite and hematite content. Dimmed signals stemming from non-applied box corrections lead to the reduced amplitude of DRS-derived goethite and hematite signals, possibly masking valuable paleoclimate information preserved in the sediment archive.

**Workflow implementation:** the third part of this contribution presents a new tool, the R package 'LESLIE' to visualize commonly measured L*, a*, b* values in RGB colors and to conduct a color-contrast enhancement in multiple enhancement cycles. Several studies measure L*, a*, b* and present this data with depth-line graphs, which are not easy to imagine as real colors. Color contrast enhancement has several advantages. First, color contrast enhancement makes subtle changes in color nuances visible, which otherwise would be invisible to the naked eye. In loess-paleosol sequences, these subtle color nuance changes might indicate millennial-scale climate change variability, which might be overlooked otherwise.

Additionally, dependent on the number of selected cycles, tephra layers (volcanic ashes) might be detected based on their diverging mineral assemblage. The mixture with bracketing loess might diminish, resulting in visible color changes but might be overseen if color contrast enhancement is not tested. Colorimetrically indicated preserved volcanic ashes can be further investigated by magnetic, mid-infrared, and granulometric properties, further strengthening the evidence for preserved tephra layers and valuable stratigraphical marker horizons.

## Ethics statements

MethodsX has ethical guidelines that all authors must comply with. In addition, we ask you to complete the relevant statement(s) below. Please delete those which are not relevant to your work.

Supplementary material *and/or* additional information

The R Package 'LESLIE' is available here: 10.5281/zenodo.7257765

## CRediT authorship contribution statement

**Christian Laag:** Conceptualization, Methodology, Software, Validation, Data curation, Writing – original draft, Writing – review & editing. **France Lagroix:** Writing – original draft, Writing – review & editing, Investigation, Supervision. **Sebastian Kreutzer:** Software, Validation, Writing – review & editing. **Stoil Chapkanski:** Methodology, Writing – review & editing. **Christian Zeeden:** Writing – review & editing. **Yohan Guyodo:** Writing – original draft, Writing – review & editing, Investigation, Supervision.

## Declaration of Competing Interest

The authors declare that they have no known competing financial interests or personal relationships that could have appeared to influence the work reported in this paper.

## Data Availability

Data will be made available on request.
